# Parental beliefs and attitudes towards child caries prevention: assessing consistency and validity in a longitudinal design

**DOI:** 10.1186/1472-6831-8-1

**Published:** 2008-01-23

**Authors:** Erik Skaret, Ivar Espelid, Marit S Skeie, Ola Haugejorden

**Affiliations:** 1 Faculty of Dentistry, UoB, PB 7800, N-5020, Bergen, Norway; 2 Faculty of Dentistry, UoO, P.b. 1142, Blindern, N-0317 Oslo, Norway

## Abstract

**Background:**

Exploring the stability of self-reports over time in observational studies may give valuable information for the planning of future interventions. The aims of the present study were: 1) to explore the consistency of parental self-reports of oral health habits, beliefs and attitudes towards child oral health care over a two-year period; 2) to evaluate possible differences in item scores and consistency between parents with different immigrant status; and 3) to assess the construct validity of items measuring parental beliefs and attitudes towards child oral health care.

**Methods:**

The sample (S1, n = 304) included parents of 3-year-old children in Oslo, Norway; 273 mothers of western origin (WN-group) and 31 of non-western origin (IM-group). They were surveyed in 2002 (child age 3 years) and in 2004 (child age 5 years). Two additional samples of parents were also included; one with 5-year old children in 2002 (S2, n = 382) and one with 3-year-old children in 2004 (S3, n = 427). The questionnaire included items measuring child oral health habits and parental beliefs and attitudes towards child oral health care.

**Results:**

In 2002, 76.8% of the parents reported that they started to brush their child's teeth before the age of 1 year. Eighty-five percent of them reported the same in 2004; 87.0% of the WN-group and 33.3% of the IM-group (*P *< 0.001). For 17 of 39 items measuring beliefs and attitudes the responses were more positive for the WN-compared to the IM-group. Parents of caries-free children in 2004 reported significantly more positive beliefs and attitudes towards child oral health care in 2002 compared to parents of children with caries in 2004 (*P *< 0.05, *P *< 0.01 and *P *< 0.001). No differences in mean item scores were found between the three samples S1, S2 and S3.

**Conclusion:**

The results showed a fair to good consistency of parental self-reports from 2002 to 2004. They also indicate that parents with different cultural backgrounds should be evaluated separately and in a cultural context.

## Background

The validity of self-reported information on health related issues is of fundamental importance for the interpretation and understanding of findings. For measurements of beliefs and attitudes the construct validity refers to whether the test reflects the underlying, individual differences explained by a theoretical model. For dental self-reports both clinical validity and high level of concordance between children and care-givers have been found [1]. However, the validity analyses are not meaningful if the self-reports intending to measure the construct are unreliable, i. e. they are not stable over time. In addition, it cannot be assumed that a measure proved to be reliable and valid in cross-sectional studies will be suitable for the purpose of detecting meaningful changes (responsiveness) in longitudinal interventions [2].

As self-reported factual information is unlikely to be influenced by other factors than lack of memory, it should be fairly consistent over time. For self-reports intending to measure beliefs and attitudes, consistency over time is more uncertain. These individual self-reports may change depending on cultural differences and environmental factors. Increased multiculturalism and language diversity is likely to create a need for better understanding of possible impacts on self-reports of beliefs and attitudes[3].

If self-reports are not reliable and stable over time, the measurement of the effects of specific interventions to change attitudes and behaviour will also be unreliable. For assessment of test-retest reliability, a few weeks is the recommended interval between tests (long enough to forget the answers and short enough to ensure stability)[4]. For intervention studies, the period between the pre- and post-measurements will often be much longer and the measurements may be influenced by inconsistencies over time, even if the instrument has shown good reliability in a short time period. Exploring the consistency/inconsistency of self-reports over time in situations without interventions, may give valuable information for the planning of future action. Consistency of self-reports may vary with cultural norms. Consequently, it may be especially important to assess both within-group variation and interaction effects between groups in ethnic-comparative research [5]. Few studies have evaluated the consistency/inconsistency of parental self-reported oral health related beliefs and attitudes in groups of parents with different immigrant status.

The aims of the present study were: 1) to explore the consistency of parental self-reporting of oral health habits, beliefs and attitudes towards child oral health care over a two-year period; 2) to evaluate possible differences in item scores and consistency between groups of parents with different immigrant status; and 3) to assess the construct validity of items measuring parental beliefs and attitudes toward child oral health care by exploring their relationship to the caries experience of the child. We hypothesized that parents whose children were caries-free at age 5 years had more positive attitudes towards child oral health care when the children were 3 years old than parents of children with caries experience at age 5.

## Methods

### Sample and design

**Figure 1:**
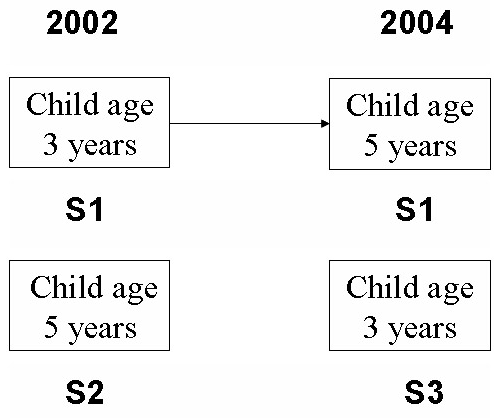
Design of the study.

The study sample included parents of 3-year-old children, drawn from 7 different dental clinics in Oslo, Norway. For further details, see Skeie et al., 2006 [6]. These parents were followed longitudinally from 2002 (child age 3 years) to 2004 (child age 5 years) (S1, n = 304). Two additional samples of parents were also included; one with 5-year old children in 2002 (S2, n = 382) and another with 3-year-old children in 2004 (S3, n = 427). The samples S1 and S2 were drawn in 2002, while sample (S3) was drawn in 2004 (Fig. 1).

### Questionnaire

The questionnaire included questions about oral health habits (start and frequency of the child's tooth-brushing and frequency of sugar snacking). Parental attitudes towards and beliefs about child oral health care were measured using a psychometric instrument developed by Pine et al. (2004) [7]. The following questions recorded the start and frequency of tooth brushing: 1) *How old was your child when he/she first started having his/her teeth brushed/cleaned *(1 = before age 1, 2 = 1–2 years of age, 3 = 2–3 years of age, 4 = after age 3, 5 = cannot remember; and 6 = does not brush his/her teeth); and 2) *How often do you brush your child's teeth? *(1 = never, 2 = not every day, 3 = once a day, 4 = twice a day, 5 = three times a day, and 6 = every second day). Frequency of sugar snacking was evaluated by the question: *How often do you give your child sweets? *(1 = every day, 2 = most days, 3 = once a week, 4 = sometimes; and 5 = never). The psychometric instrument measuring parental attitudes and beliefs related to oral health of the child had been constructed, pre-tested for reliability and validity and used in a collaborative international multi-centre study[7], and also in publications based on the same material. The study protocol was approved by the Regional Committees for Medical Research Ethics and the Norwegian Data Inspectorate. Written informed consent to participate in the study was obtained from the parents. For further details, see Skeie et al., 2006 [6]. We have used the original items including the differences in direction of the scores (from strongly disagree to strongly agree or vice versa) (Tables 1, 2, 3).

**Table 1:** Means (SD) for single items with significant group (G), time (T) (2002 – 2004) or interaction effect (I).

**Item**		**WN-group**	**IM-group**	
		Mean (SD)	Mean (SD)	F-value

C18 As a family, we intend controlling how often our child has sugary foods or drinks between meals (1 = strongly disagree, 5 = strongly agree)	2002 2004	4.19 (0.81) 4.30 (0.70)	3.88 (0.74) 4.00 (0.72)	G: 5.36* T: 1.78 I: 0.00
C23 It is worthwhile to give our child sweets/biscuits to behave well (1 = strongly agree, 5 = strongly disagree)	2002 2004	4.44 (0.79) 4.54 (0.80)	3.67 (1.01) 3.67 (1.17)	G: 38.49*** T: 0.23 I: 0.23
C25 The people we know well would feel it was important to control how often our child has sugary foods and drinks (1 = strongly disagree, 5 = strongly agree)	2002 2004	2.62 (1.16) 2.90 (1.19)	3.46 (1.14) 3.71 (0.96)	G: 15.98*** T: 3.55 I: 0.01
C26 In our family, it would be unfair not to give sweets to our child every day (1 = strongly agree, 5 = strongly disagree)	2002 2004	4.44 (1.05) 4.59 (0.83)	3.50 (1.35) 3.50 (1.35)	G: 36.80*** T: 0.33 I: 0.33
C27 It is often too stressful to say no to my child when they want sweets (1 = strongly agree, 5 = strongly disagree)	2002 2004	4.10 (0.97) 4.35 (0.82)	3.25 (1.03) 3.29 (1.30)	G: 33.25 *** T: 1.98 I: 0.96
C30 It is not worth it to battle with our child to brush his/her teeth twice a day (1 = strongly agree, 5 = strongly disagree)	2002 2004	4.39 (0.86) 4.58 (0.76)	3.67 (1.13) 3.58 (1.41)	G: 35.12*** T: 0.21 I: 1.50
C32 The dentist is the best person to prevent tooth decay in our child (1 = strongly agree, 5 = strongly disagree)	2002 2004	4.00 (0.82) 4.22 (0.80)	3.29 (1.00) 3.54 (1.18)	G: 24.05*** T: 4.86* I: 0.01

* *P *< 0.05, ** *P *< 0.01, *** *P *< 0.001.

**Table 2:** Means (SD) for single items with significant group (G), time (T) or interaction effect (I) (2002–2004).

**Item**		**NW-group**	**IM-group**	
		Mean (SD)	Mean (SD)	F-value

A4 As a family, we are confident that we can reduce the chances of our child getting tooth decay (1 = strongly disagree, 5 = strongly agree)	2002 2004	4.58 (0.61) 4.68 (0.63)	4.12 (0.83) 4.20 (0.81)	G: 18.05*** T: 1.23 I: 0.00
A5 Tooth decay will not get better by itself (1 = strongly disagree, 5 = strongly agree)	2002 2004	4.81 (0.56) 4.78 (0.57)	4.40 (0.81) 4.56 (0.58)	G: 9.91** T: 0.88 I: 1.87
A9 As parents, it is our responsibility to prevent our child getting tooth decay (1 = strongly disagree, 5 = strongly agree)	2002 2004	4.82 (0.43) 4.85 (0.36)	4.40(0.87) 4.64(0.57)	G: 19.08*** T: 5.94* I: 3.62
A12 If our child does not want to brush his/her teeth every day we don't feel we should make them (1 = strongly agree, 5 = strongly disagree)	2002 2004	4.35 (0.92) 4.50 (0.89	3.42 (1.52) 3.53 (1.38)	G: 45.17*** T: 0.34 I: 0.34
A18 As a family, we intend brushing our child's teeth for him/her (1 = strongly disagree, 5 = strongly agree)	2002 2004	4.79 (0.45) 4.79 (0.54)	4.42 (0.59) 4.23 (1.23)	G: 17.57*** T: 0.09 I: 0.09
A19 We intend brushing our child's teeth for him/her twice a day (1 = strongly disagree, 5 = strongly agree)	2002 2004	4.36 (0.88) 4.57 (0.72)	4.08 (1.02) 4.08 (1.11)	G: 5.70* T: 0.50 I: 2.48
A23 I don't know how to brush my child's teeth properly (1 = strongly agree, 5 = strongly disagree)	2002 2004	4.18 (0.86) 4.46 (0.75)	3.92 (0.91) 3.48 (1.33)	G:20.53*** T: 0.50 I: 10.78***
A25 If our child uses a fluoride toothpaste, it will prevent tooth decay (1 = strongly disagree, 5 = strongly agree)	2002 2004	4.09 (0.81) 4.27 (0.74)	3.92 (0.74) 3.63 (0.93)	G: 6.47* T:0.50 I: 3.35
A30 It would not make any difference to our child getting tooth decay, if we helped him/her brush every day (1 = strongly agree, 5 = strongly disagree)	2002 2004	4.55 (0.62) 4.50 (0.74)	3.88 (0.97) 3.70 (1.26)	G: 34.95*** T: 1.24 I: 0.42
A33 We cannot make our child brush his/her teeth twice a day (1 = strongly agree, 5 = strongly disagree)	2002 2004	4.22 (0.99) 4.46 (0.85)	3.92 (1.13) 3.93 (1.17)	G: 7.90** T: 1.30 I: 1.30

* *P *< 0.05, ** *P *< 0.01, *** *P *< 0.001.

**Table 3:** Attitude items with significant differences in mean (SD) scores in 2002 according to caries (dmft > 0) or caries free (dmft = 0) in 2004.

**Item**	**Caries 2004**		
	dmft = 0	dmft > 0	
	Mean (SD)	Mean (SD)	F-value

A4 As a family, we are confident that we can reduce the chances of our child getting tooth decay (1 = strongly disagree, 5 = strongly agree)	4.60 (0.61)	4.30(0.75)	9.85**
A10 Our child losing a baby tooth due to tooth decay would be upsetting (1 = strongly disagree, 5 = strongly agree)	4.26 (0.96)	3.47 (1.12)	27.13***
A25 If our child uses a fluoride toothpaste, it will prevent tooth decay (1 = strongly disagree, 5 = strongly agree)	4.12 (0.81)	3.87 (0.73)	4.43*
C18 As a family, we intend controlling how often our child has sugary foods or drinks between meals (1 = strongly disagree, 5 = strongly agree)	4.22 (0.80)	3.90 (0.80)	6.68**
C23 It is worthwhile to give our child sweets/biscuits to behave well (1 = strongly agree, 5 = strongly disagree)	4.43 (0.77)	4.13 (1.05)	5.31*

* *P *< 0.05, ** *P *< 0.01, *** *P *< 0.001.

### Consistency

Consistency in self-reporting (single question and mean individual item scores for the psychometric instrument) was evaluated by comparing responses from the same parents in 2002 and 2004 (S1), including a comparison between: 1) the immigrant group (IM-group; mother of non-western origin: Eastern Europe, Asia, Africa, Turkey, South and Central America) and 2) the western native group (WN-group; mother of western origin: Western Europe, North America, Australia and New Zealand). The mean individual item scores were also compared with the corresponding scores from the two independent samples of parents (S2 (2002) and S3 (2004)) (Fig. 1).

### Validity

The instrument had previously been translated into Norwegian and translated back into English by a bilingual person [6]. Construct validity for items measuring attitudes and beliefs related to oral hygiene and diet was evaluated by assessing the relationship between these variables and self-reported tooth brushing habits (start and frequency) as well as the frequency of sugar snacking. The responses were dichotomized as *agree *(agree and strongly agree) vs. *disagree *(disagree and strongly disagree). The response alternative *neither agree nor disagree *was not included. The frequency of tooth brushing was dichotomized as *twice a day or more *(responses 4 and 5) vs. *less than twice a day *(responses 1, 2, 3 and 6). Frequency of sugar snacking was dichotomized as *once a week or more seldom *(responses 3, 4 and 5) vs. *more frequently than once a week *(1 and 2). Furthermore, we evaluated the relationship between the parental attitudes and beliefs related to child oral health care in 2002 (child age 3 years) and the child's caries prevalence in 2004 (child age 5 years), by comparing parents of children with caries at d_3–5_mfs-level with parents of caries-free children in 2004 (Table 3). Caries-free was defined as children without diagnosed caries into dentin.

### Caries registration

Seven trained and calibrated dental hygienists undertook the dental examinations in 2002 and 2004. A detailed caries diagnostic system was applied, using five severity grades from outer enamel to inner dentin. The two incipient grades of caries (1 and 2) were denoted enamel lesions and the others dentin lesions. For more details, see Skeie et al, 2006 [6].

### Data analyses

The Statistical Package for Social Sciences (SPSS^®^, version 14.0 for PC) was used for the analyses. Differences between groups were assessed using One-Way ANOVA and Chi-square (cross tabulation). To explore the difference between groups (WN-group vs. IM-group), General Linear Model (repeated measures analysis of variance) was used with *group *(WN vs. IM) and *time *(2002 and 2004) as factors. A significant time and/or interaction effect was further explored by use of t-tests. Based on the distribution of the data the results were confirmed by additional non-parametric analyses. In the test-retest analyses (reliability analyses for scales) an intraclass correlation coefficient (ICC) between 0.40 and 0.75 was considered fair to good consistency [8].

## Results

### Oral health habits

#### Start of tooth brushing

In 2002, 76.8% (232/302) of the parents reported that they started to brush their child's teeth before the age of 1 year; 81.7% of the WN-group and. 34.5% of the IM-group (χ^2 ^= 33.14, *P *< 0.001). Eighty-five percent (197/232) of them reported the same in 2004; 87.0% of the WN-group and 33.3% of the IM-group (χ^2 ^= 19.44, *P *< .001). The percentage agreement for the whole group from 2002 to 2004 was 77%. Test-retest analyses for the responses to the question *How old was your child when he/she first started having his/her teeth brushed/cleaned *(2002 and 2004) showed a fair to good consistency, with an ICC of 0.52 (CI: 0.40–062).

#### Start of tooth brushing and attitudes toward oral hygiene

Of the parents who in 2002 reported that they started to brush their child's teeth before 1 year of age, 96.1% (223/232) disagreed (disagree or strongly disagree) with the statement: (C30)" *It is not worth it to battle with our child to brush his/her teeth twice a day*", compared with 84.5% of the parents who reported that they started to brush their child's teeth later than age one (χ^2 ^= 10.81, *P *< 0.001).

#### Frequency of tooth brushing

The frequency of parents who reported brushing their child's teeth twice a day or more often increased from 64.9% in 2002 to 81.6% in 2004 (χ^2 ^= 67.24, *P *< 0.0001) (no differences between groups). Their percentage agreement for brushing twice a day or more often vs. less than twice a day from 2002 to 2004; was 77%. The ICC for frequency of tooth brushing was 0.53 (CI: 0.41–0.62).

#### Frequency of tooth-brushing and attitudes toward oral hygiene

Of the parents who reported brushing the child's teeth twice a day or more often in 2002, 90.4% (178/197) disagreed with the statement (C30) "*It is not worth it to battle with our child to brush his/her teeth twice a day*", and 98.2% (168/171) of them (n = 171) also disagreed with this statement in 2004. The percentage agreement for the individual responses from 2002 and 2004 was 94.0%, and the test-retest analyses showed a fair to good consistency for the responses to this item, ICC = 0.50 (CI: 0.37 – 0.60).

#### Frequency of sugar snacking

In 2002, 87.2% (265/297) of the sample reported giving their child sweets only once a week or occasionally, and 93.5% (245/262) of them reported the same in 2004. In 2002 a total of 93.8% (256/273) disagreed (disagree or strongly disagree) with the statement (C26) *"In our family it would be unfair not to give sweets to our child every day"*. As shown in Table 1, this attitude showed a good consistency from 2002 to 2004 for both the WN- and the IM group, but there was a significant group difference (F = 36.8, *P *< 0.001).

#### Attitudes toward sugar snacking

A good validity was found for the statement *"In our family, it would be unfair not to give sweets to our child every day" *(Item C26). Of parents who reported to give their child sweets only once a week or occasionally, respectively 90.6% (231/255) and 92.7% (243/262) disagreed (disagree or strongly disagree) with this statement, in 2002 and in 2004. The percent agreement was 89.0%, and the ICC for the test-retest of this item was 0.50 (CI: 0.37 – 0.60).

### Differences in item scores between groups in 2002 and 2004

**Figure 2:**
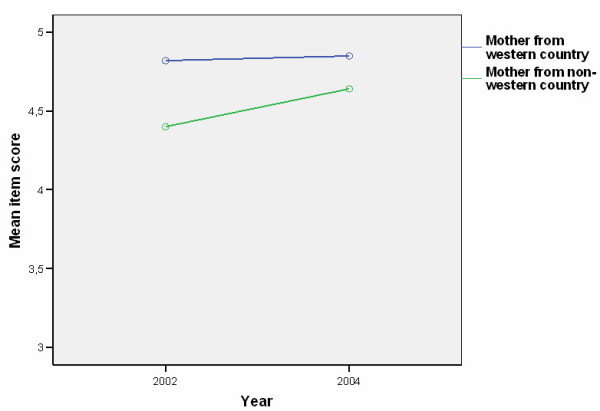
(A9) Distribution of parents according to group and responses to the statement *"As parents it is our responsibility to prevent our child getting tooth decay" *in 2002 (1) and 2004 (2) (1 = strongly disagree, 5 = strongly agree).

**Figure 3:**
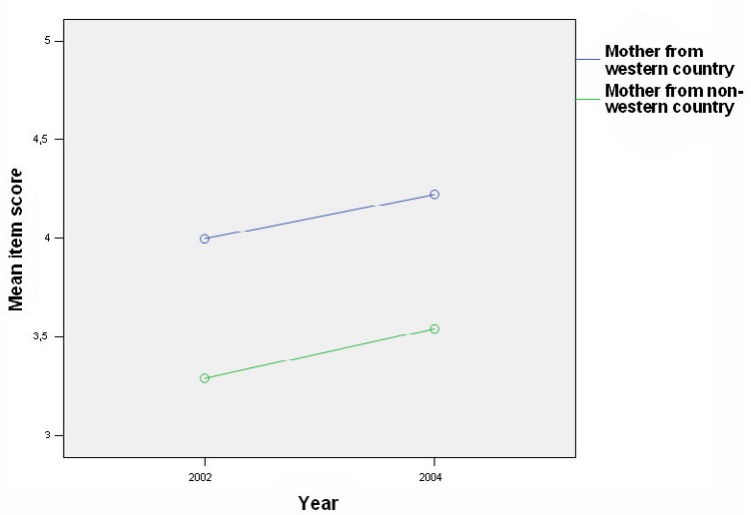
(C32) Distribution of parents according to group and responses to the statement "*The dentist is the best person to prevent tooth decay in our child" *in 2002 (1) and 2004 (2) (1 = strongly agree, 5 = strongly disagree).

**Figure 4:**
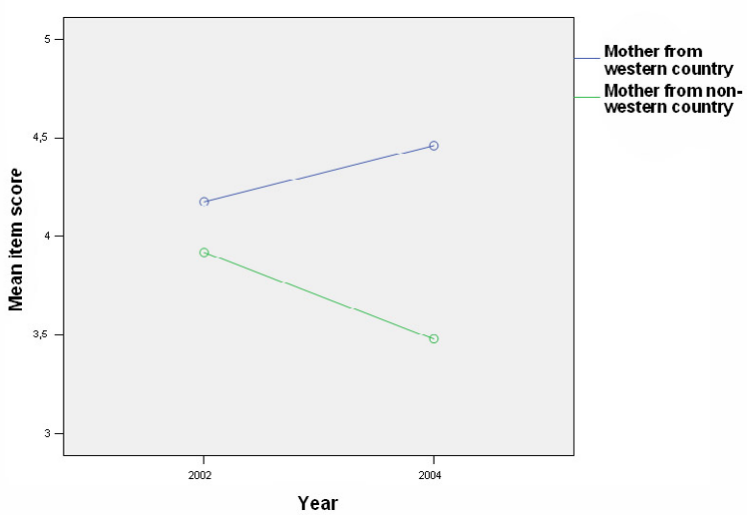
(A 23) Distribution of parents according to group and responses to the statement "I don't know how to brush my child's teeth properly" (1 = agree, 5 = disagree).

Mean scores were calculated for 39 different items measuring beliefs and attitudes towards child oral health care. Items showing differences between groups (WN-group (n = 273) and the IM-group (n = 31)), are presented in Tables 1 and 2. For 17 of the 39 items (44%) there were significant differences either in mean scores between groups (more positive attitudes for the NW-group), in time (from 2002 to 2004), or interaction effect (group changes in different directions or change over time for one group only). A time effect was found for only two items: A9 (*As parents, it is our responsibility to prevent our child getting tooth decay*) (Table 2, Fig. 2) and C32 (*The dentist is the best person to prevent tooth decay in our child*) (Table 1, Fig. 3). When looking at each group separately, the difference from 2002 to 2004 was only statistically significant for C32, and only for the WN-group (t = 4.0, *P *< 0.001). For item A23 (*I don't know how to brush my child's teeth properly*) there was a difference both between groups and an interaction effect (increase in score for the WN-group, and a decrease for the IM-group) (Table 2 and Fig. 4.). T-tests showed that the difference between groups was significant only in 2004 (t = 4.8, *P *< 0.001), and that the change over time for the WN-group from 2002 to 2004 (more confident in 2004 compared to 2002) was significant (t = 4.6, *P *< 0.001). The tendency towards a decrease in confidence from 2002 to 2004 in the IM group was not statistically significant.

#### Comparisons between independent samples

Comparisons of mean item scores in sample S1 in 2002 with responses to the same questions from two independent samples of parents; S2 (data collected in 2002) and S3 (data collected in 2004) (Fig. 1) showed no significant differences between the samples.

### Beliefs and attitudes in 2002 and the relationship to caries experience in 2004

The differences in mean item scores of the parental self-reports in 2002 related to caries experience (children with caries at d_3–5_mfs-level and caries-free children) in 2004 are shown in Table 3. Parents of caries-free children in 2004 had significantly more positive responses (2002) than parents of children with caries in 2004 to the following statements: "*As a family, we are confident that we can reduce the chances of our child getting tooth decay," "Our child losing a baby tooth due to tooth decay would be upsetting,"* "*If our child uses a fluoride toothpaste, it will prevent tooth decay," "As a family, we intend controlling how often our child has sugary foods or drinks between meals" *and *"It is worthwhile to give our child sweets/biscuits to behave well."*

## Discussion

The major purpose of the present non-intervention study was to assess the general consistency and validity of parental self-reporting related to child oral health care in a two-year period. Generally, the results showed a fair to good stability of the parental self-reporting from 2002 to 2004 [8], but indicated that it may differ between groups with different cultural backgrounds. We found good construct validity for items intending to measure parental beliefs and attitudes towards child oral health care.

The strength of our study was its longitudinal design, providing both parental self-reporting and caries registration when the child was 3 and 5 years of age. In addition we had the opportunity to compare the study sample with the responses from two independent samples. Even if the study sample was not representative of the population, the participants came from different socioeconomic and ethnic backgrounds from 16 countries on different continents. The small number of subjects in the IM group is a limitation; consequently the comparisons between groups should be interpreted with caution.

We found that self-reports reflecting objective answers (age when starting to brush the child's teeth) also had some degree of inconsistency over a two-year period. The results indicated no differences in the consistency for these self-reports compared to items measuring beliefs and attitudes. Two years is a long period for a test-retest design. Some self-reports are probably influenced by lack of memory, which would influence the stability of the responses in a negative way. Even in the absence of interventions, attitudes and beliefs may change over a two year period. The comparisons between independent samples collected at different times were valuable. The result showing no differences in mean item scores between the samples S1,2 versus S3 (recruited 2 years later) may indicate that no campaigns have influenced the stability of the self-reports. However, we have no information about how local campaigns may have influenced individuals or sub-groups during the 2-year period. It is reasonable to expect that a test-retest period of two years gives lower ICC values than if carried out with an interval of only a few weeks [9].

The significant differences between parents of children with caries and parents with caries-free children (age 5) indicated good validity for the items measuring attitudes and beliefs related to caries prevention (Table 3). This result confirms the good validity found in the original study where the items were developed and tested [10], a study which also found that beliefs about the importance of tooth-brushing were very strong among Norwegian parents [10]. The clinical validity of parental self-reporting confirmed our hypothesis, and the results reported by Jamieson et al. [1]. This result thereby supports their suggestion that good validity makes self-reporting a convenient method for collecting dental health information about children[1]. The fact that parental attitudes and beliefs are good predictors of future dental health of the child also support the fact that interventions to change the parental beliefs and attitudes towards child caries prevention may be a reasonable approach in preventive oral health care.

The consistency found for the self-reported habits (frequency of tooth brushing and intake of sweets) over the two year period indicates that habits established at 3 years of age tend to persist during the next two years. This is important, since these variables have been shown to be related to caries in children[11]. The habits should therefore be established at as early an age as possible [12].

The differences in mean item scores between groups and the tendency of differences in consistency, may justify separate subgroup evaluation, based on criteria like socio-economics, oral health of the child and differences in cultural background.

## Conclusion

The strong relationship found between items measuring beliefs and attitudes towards child oral health care and the caries status of the child two years later makes self-reporting a convenient method for obtaining dental health information about children. However, the effect of future interventions aimed at changing parental attitudes and behaviour has to be adjusted for some degree of inconsistency of self-reporting over time. Sub-groups of parents with different cultural backgrounds should be evaluated separately and findings interpreted accordingly. Differences between groups may also require alternative preventive strategies for sub-groups of parents.

## Competing interests

The author(s) declare that they have no competing interests.

## Authors' contributions

IE conceived and designed the study. MSS collected the data. ES performed the statistical analyses, the interpretation of the data and the manuscript drafting. OH assisted in the interpretation of the data, the manuscript drafting and the copyediting of the final version. All the authors have participated actively in the writing of the manuscript and have read and approved the final version.

## Pre-publication history

The pre-publication history for this paper can be accessed here:


